# Synthesis of Repair Materials and Methods for Reinforced Concrete and Prestressed Bridge Girders

**DOI:** 10.3390/ma13184079

**Published:** 2020-09-14

**Authors:** Azin Ghaffary, Mohamed A. Moustafa

**Affiliations:** Department of Civil and Environmental Engineering, University of Nevada, Reno, NV 89557, USA; aghffary@nevada.unr.edu

**Keywords:** bridge girder repair, FRP-based materials, shear cracks, overheight vehicle collision, EB repair approach, NSM repair approach, anchorage system

## Abstract

Bridge structures nationwide across the United States are aging and in need of repair or, in some cases, full replacement. Repair decisions are common among bridge owners because of the economic feasibility when compared to the higher cost of full replacement of damaged or deteriorated bridge components such as girders. Using a proper repair approach, as a long-term or just a short-term solution, can lead to benefits that could not be achieved otherwise such as considerable savings in both time and cost. Additionally, an appropriate repair approach can help avoid adverse environmental impacts, interruptions to service, overburdening of nearby infrastructure, and local opposition to construction. The main objective of this paper is to provide a synthesis of the repair methods and materials for reinforced concrete bridge girders proposed in research studies, i.e., state-of-the-art as well as state-of-the-practice established methods. Different steps in the general repair procedure are explained first. Next, a detailed description of three common bridge girder deficiencies, i.e., shear, flexural, and fire damage, is provided. For each damage type, the main causes and common solutions found in the literature are presented. The authors then provide specific recommendations to each repair procedure. This is intended to enable researchers, engineers, and decision makers to compare the available repair methods more conveniently to find the optimal repair approach for specific projects based on economic and environmental requirements as well as structural and construction conditions.

## 1. Introduction

A majority of the United States’ transportation infrastructure is over 50 years old [1]. Among the bridge structures, approximately 30% of more than 607,000 bridges and 23% of 163,000 single-span concrete bridges in the country are currently classified as either structurally deficient or functionally obsolete. The former is described as a bridge with deficiencies such as corroded elements that need to be repaired. The latter, however, can be referred to as a bridge that has inconsistencies with the current code requirements, such as narrow shoulders or lane widths, or inadequate clearance for oversize vehicles [2,3]. Common sources of damage to bridge girders are any of the following reasons or combinations of them [1,4,5,6,7,8,9,10,11,12,13,14]: (1) chloride attack, corrosion, and deterioration; (2) fatigue damage accumulation; (3) accidental damage such as overheight vehicle impact; (4) upgraded loading requirements and more stringent assessment codes; and (5) initial design flaws, construction defects, and a lack of maintenance.

The available options applicable to a bridge with damaged girders are “leave and monitor”, “repair”, or “replacement” of the girders. Harries et al. [15] classified bridge girder damage intensities into minor, moderate, and severe levels. Each intensity and the corresponding effects on the member’s capacity as well as the required repairs are reported in Table 1. Replacing bridges can cause economical loss and inconvenient vehicle traffic [16], and is usually a more expensive option compared to repair [17]. Repair costs of a prestressed I-girder range from 35% to 69% of the cost of the superstructure replacement [18]. Additionally, it can cause environmental impacts, interruptions to service, overburdening of nearby infrastructure, and local opposition to construction [8]. Studies indicate that average girder replacement costs about USD 8000 per ft of girder which is very expensive [19]. Accordingly, in certain projects, retrofitting is the only option because of budgetary restrictions that bridge owners are facing [20]. However, assessment and strengthening of deficient bridges in the United States has been estimated as being in excess of USD 140 billion [8], which is still a huge amount of money. These factors make the repair and strengthening of bridge structures a crucial topic concerning all nations, which should be done efficiently and in an economic way. Some of the important factors in evaluating a proper repair method are safety, repair time, and economy [6]. Otherwise, in the absence of an economical and efficient repair technique, the bridge should be considered deficient. This is the case for one in nine of the nation’s bridges that are classified as deficient due to a lack of funding for maintenance and repair, environmental degradation of structural components, and/or increased vehicular weight. In order to eliminate the bridge deficient backlog by 2028 in the United States, USD 20.5 billion would need to be invested annually [1]. In practice, most of the repair methods might cause concerns for the industry and Departments of Transportation (DOTs) decision makers regarding their performance in effectively strengthening the deficient bridge girders. This is because for most repair techniques, there is a lack of readily available laboratory results.

The main objective of this study is to gather the information about different materials and methods of bridge girder repair implementation that have been used so far in practice or merely proposed through research projects. More than 200 studies have been reviewed and summarized here with a focus on the repair of reinforced concrete (RC) bridge girders which contribute to more than 60% of the bridge inventory in the US [21]. For this purpose, the steps required for the general repair procedure of damaged RC bridge girders, regardless of the damage type, are explained. Next, each girder deficiency, i.e., shear, flexural, and fire damage, is addressed in a separate section. For each damage type, the causes and the main solutions found in the literature are first described. Then, recommendations for the repair procedure specific to that damage type are proposed by the authors based on surveying and ranking the frequency of use of the different methods. This is meant to enable researchers, engineers, and decision makers to compare the available repair methods more conveniently to find the optimal repair approach for specific projects based on economic and environmental requirements as well as structural and construction conditions.

## 2. General Repair Procedure

The main steps in a typical repair procedure can be summarized in eight steps: (1) inspection and monitoring; (2) making the first decision on choosing a repair material; (3) making the second decision on choosing the repair method; (4) surface preparation; (5) repair design (6) application of repair material; (7) prestressing of repair material, if applicable; (8) anchorage system; and (9) strand splicing, if needed. A summary of the state-of-the-art and practice on each of the eight steps is presented next.

### 2.1. Inspection and Monitoring

This may be performed on a periodic or usage basis, or motivated by reports of damage or extreme loading to determine the severity of the damage, cause, and prognosis [22,23]. The existing load-carrying capacity of the structure should be determined. Any structural deficiencies and their causes should be identified. The condition of the concrete substrate should also be understood. Other parameters that should be specified as well include: the existing dimensions of the structural members; the location, size, and causes of cracks and spalls; the location and extent of any corrosion of reinforcing steel; the presence of any active corrosion; the quality and location of existing reinforcing steel; the in-place compressive strength of the concrete; and the soundness of the concrete, particularly the concrete cover in all areas where the strengthening material is going to be bonded to the concrete. Then, a decision is made about the type of action needed for the bridge which can be: repair, demolish, or leave alone and keep monitoring [4,24]. In making this decision, the responsible authorities will consider the cost and durability of the repair compared to demolishment or replacement.

### 2.2. Decision #1: Choosing a Repair Material

If repair is needed, then the next step is to choose an appropriate repair material. Availability and durability of the material, ease of handling on site, cost-effectiveness, type and condition of the structural element, and the targeted enhancement in the structure are factors that should be considered in making this decision [25]. Common materials used for the repair of RC bridge girders are as follows.

#### 2.2.1. Fiber-Reinforced Composites (Since 1980s)

**Figure 1:**
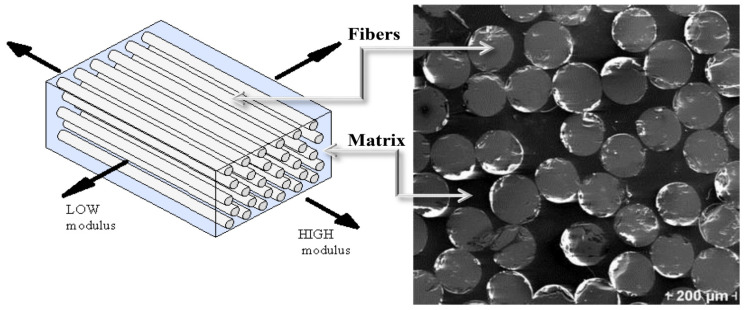
Schematic of fiber-reinforced composites [1].

Fiber-reinforced composites, as shown in Figure 1, are a combination of two different materials (i.e., the reinforcing fibers and the matrix). The type of matrix and fiber, orientation of the fibers, as well as the ratio of matrix to fiber content will affect the properties of the resulting composite [1]. Strengthening of concrete members using fiber-reinforced composites, initially used for aerospace applications, started in the mid-1980s, and it has gained popularity in recent years, especially for bridge repair applications, due to their superior characteristics such as: high strength-to-weight ratio, anti-corrosive properties, high tensile strength, insect and fungal resistance, low thermal conductivity, ease of installation, and flexibility in application. Fiber-reinforced composites are about 85% to 73% lighter than the steel. This means an ease of handling and less equipment and workforce requirements on site. Despite their higher cost compared to steel, they are usually the preferred strengthening approach for long-term repair projects due to their superior characteristics listed above. This is while steel materials with proper corrosion treatment might be a better choice for short-term retrofit projects [19,25,26,27,28,29,30,31,32].

Depending on the type of the matrix used, fiber-reinforced composites are categorized into three groups: polymeric composites, cement-based composites, and hybrid composites.

- Polymeric composites: Polymeric composites (also called fiber-reinforced polymers or FRP) are fibers embedded in a polymeric resin such as unsaturated polyester, epoxy, vinylester, phenolic, and polyurethane resins. Epoxy resins are the most common matrix in structural repair applications due to their characteristics such as good adhesive properties, low shrinkage during curing, and resistance to environmental degradation [1,33]. Popular fibers used are steel, basalt, carbon, and glass, which result in SFRP, BFRP, CFRP, and GFRP composites, respectively. The fibers and the resin work as a system together; the fibers provide load carrying capacity, high tensile strength, and rigidity, while the resin protects and transfers the load to the fibers and works like a binder to them [28,33,34]. However, one of the major limitations in using FRP materials as a repair or strengthening approach is the ductility of the element. The ductility of the concrete members strengthened with FRP materials decreases with the amount of FRP used. Therefore, a strengthening limit approach is often suggested in guidelines and codes in order to restrict the use of high amounts of FRP [35].
- Cement-based composites: Use of fibers with cementitious matrix instead of epoxy resin was proposed in the early 1980s, but did not gain that much attention until the late 1990s. Cementitious matrix has several advantages over conventional epoxy resins in terms of fire resistance, performance under ultraviolet (UV) radiation, permeability, thermal reversibility, and cost. Additionally, having similar mechanical, chemical, and physical properties as the concrete substrate is another advantage. Unlike the FRP installation safety requirement because of the characteristics of the used resins, installation of cementitious matrix can be done merely by simple troweling techniques and protective equipment for typical concrete applications. Further, unlike FRP, cementitious matrix can be applied to wet surfaces, and thus the project will not be affected by weather conditions as much. The different types of cement-based composites include sprayed concrete, textile-reinforced mortar (TRM), textile-reinforced concrete (TRC), fiber-reinforced concrete (FRC), fiber-reinforced cementitious mortar (FRCM), and mineral-based composites (MBC) [7,19,25,28,33,36,37,38]. However, one of the major concerns of such material (e.g., FRCM) is unknown long-term fatigue performance. Thus, FRCM should not yet be used as a repair alternative to bridges with a considerable level of damage and/or high traffic volume [33].
- Hybrid composites: Different fiber-based composites can be combined together as a hybrid to enhance the strengthening efficiency. For example, they can provide a progressive failure pattern to compensate for the loss of ductility that is observed in traditional fiber-reinforced materials [25]. Additionally, fiber-reinforced materials remain elastic up to failure. This is while hybrid materials can experience yielding as in steel. The modulus of elasticity of the fiber-reinforced material can be low or high depending on the fiber and the matrix utilized [25,38]. Examples of such material are CFRP rod panels (CRPs), CFRP-honeycomb (referred to as H-Lam-C), and GFRP-honeycomb (referred to as H-Lam-G) composites [39,40,41].

#### 2.2.2. Steel (Since the 1960s)

Using steel plates for the strengthening of concrete elements started in the 1960s as a fast and economical solution compared to the demolition of the structure. Ever since, it has been traditionally used for the strengthening of structural concrete members in different forms such as rods, bars, tendons, plates, strand splice systems, and steel jackets. The main benefit of steel material is its well-known properties. Nonetheless, using steel comes with the common major concerns on poor corrosion resistance and difficulty in handling on site [25,29].

#### 2.2.3. Other Materials

Other less common or emerging materials used for the repair of RC bridge girders are ultra-high-performance fiber-reinforced concrete (UHPFRC or just UHPC), aluminum alloy, ferrocement, shotcrete, innovative concrete mixes made with waste material, and coatings and sealers. Each of these materials has been used in a number of repair projects due to their specific benefits and the availability of the material [18,25,29,42,43,44,45].

### 2.3. Decision #2: Choosing a Repair Method

After the repair material is chosen, the next decision is to choose a proper way for the application of the material to the damaged girder. There are several factors affecting this decision [25,46,47]: (1) whether the repair technique is commercially available, and (2) girder type (box girder or I-girder), where the shape of the girder cross-section is important in the choice of the repair technique. For example, for rectangular beams, the most common way of repair is fully wrapping the member, which is, on the other hand, impractical for T-beams due to the presence of the flange. Other factors include: (3) dominant repair limit state, (4) severity of the damage that can be repaired using a given material, (5) fatigue performance, (6) whether strengthening is needed beyond undamaged capacity, (7) whether the method can be combined with strand splicing, (8) speed of mobilization, (9) constructability, (10) whether specialized labor is required, (11) whether proprietary tools are required, (12) whether lift equipment is required, (13) how much the closure below the bridge will be, (14) time for typical repair, (15) environmental impact of repair process, and (16) durability. Another factor to consider is: (17) the resulting change in the size of the element that is being repaired as it affects the overall aesthetics of the element and might enforce additional labor cost and disruption of the structure’s service. This is controlled by the thickness of the strengthening material used. Two last but crucial considerations are: (18) cost and (19) aesthetics. Several methods are available for the application of the repair material to the damaged girder, which are summarized as follows.

#### 2.3.1. Externally Bonded (EB) Techniques

This is the most popular method for the strengthening of RC beams. In this method, the strengthening material is attached to the external surface of the beam using an appropriate adhesive material or mechanical fastener. Externally bonded FRP wraps are currently used by 24 highway departments in the US, and several other states are in the process of adopting it [40,48,49]. One of the advantages of this method over other techniques is that there is no need to remove concrete parts or drill into the section, hence an ease of application and less risk of exposing or damaging the existing reinforcement [8]. Another benefit is that it provides protection for the patch concrete and the reinforcing steel from an ingress of water and salts and thus corrosion and deterioration. Otherwise, the repairs implemented by the concrete patch alone are prone to crack under the combination of shrinkage and service loadings [33]. However, the performance of the strengthened element is highly dependent on the bonding between the concrete and the strengthening material. This is specifically important for FRP-strengthened elements where debonding failure occurs at an effective strain much lower than the ultimate strain that can be achieved by the FRP composite materials. Therefore, the full capacity of the FRP is not used. Additionally, the failure would be in a brittle manner. In order to effectively use the EB technique, the debonding failure mode should be overcome [16,50,51,52]. This method also has low fire resistance and high vulnerability to vehicle collisions [53]. The saturating resins in the EB techniques can be adversely affected by UV light over time. Moreover, their characteristics degrade when exposed to high temperatures. To partially overcome this issue, protective coatings can be applied to limit the exposure to UV light and to also provide some fire protection [19]. The aforementioned issues with the EB method have shifted the attention to other methods such as the near-surface mounted method explained next.

#### 2.3.2. Near-Surface Mounted (NSM) Techniques

**Figure 2:**
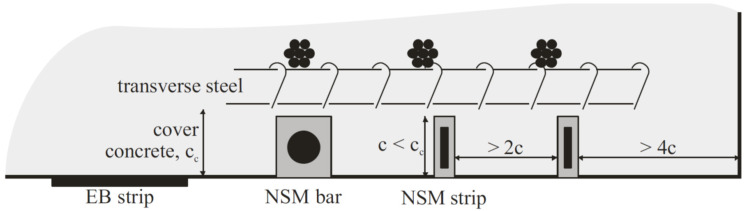
Schematic of EB and NSM bar and NSM strips for the strengthening of reinforced concrete (RC) members [46].

This method was initially presented in 1940. It is a construction technique that embeds FRP bars in the concrete surface to improve the performance of the RC structure. Although initially steel cables were used as part of the strengthening process, later on they were replaced with FRP materials due to the corrosion of steel. The FRP material is typically used in the form of bars with a rectangular cross-section (strips) or circular cross-section (bars), manufactured using the pultrusion process. Bars can be sandblasted or deformed, but studies have indicated that deformed bars have a better bond performance. Further, it has been demonstrated by some researchers that strips can lead to a more effective repair since they provide an increased surface area between the FRP and adhesive interface, with strips failing in tension rupture and achieving full composite action with the concrete [1,19,54,55]. Figure 2 illustrates the NSM technique as compared to the EB technique. The two main advantages of this method are: (1) higher bond strength can be achieved compared to the EB method since the repair material is completely enclosed in epoxy, which means a larger surface area is bonded, (2) requiring less material use due to the enhanced bond behavior [46].

The NSM method improves the fire resistance and impact damage resistance of the strengthened member compared to the EB method since the repair material is placed inside of the concrete instead of being exposed on the concrete surface, and also as a result of the increased contact area. Additionally, there is no change of dimensions in this technique, keeping the cross-section of the girder the same [28,50,54]. However, Jones [19] indicated that although this technique leads to decreased material use, the increased labor cost might offset the savings in the material cost. An example is cutting in grooves overhead which is difficult to implement for the workers. From a different perspective, Sobieck, Atadero [1] indicated that the additional time and effort that is spent on surface preparation for making the grooves will usually be compensated with the increase in the flexural properties in case the NSM rods are pretensioned.

In the case of overheight vehicle collision and damage to the prestressed girder strands, NSM might not be the best repair choice. This is because the thickness of the strand splicing equipment used for repairing the prestressing strands can conflict with cutting grooves into the concrete surface in particular locations [19]. Additionally, although the bond behavior is improved in the NSM technique, the effectiveness of the method is affected by the amount of material that can be used for the repair. The minimum spacing between the grooves that the repair materials are placed in is one example of the reasons that limit the amount of material that can be applied to the structure in this method. The NSM method is most effective when it is used in the negative moment region of a structure, so that it can remain protected from wear and abrasion. It is not recommended to be used in the positive bending region of the structure [56]. The NSM method is also sensitive to the amount of concrete cover and is not a viable option when cover is not sufficient [46].

#### 2.3.3. Embedded Reinforcement

**Figure 3:**
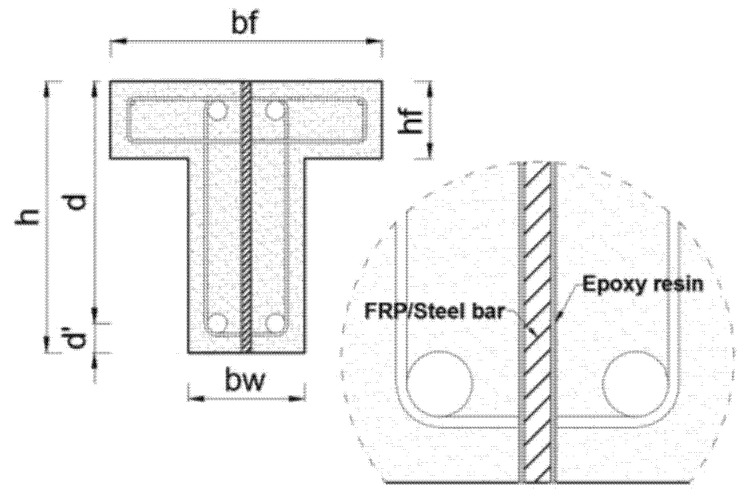
Schematic of the deep reinforcement embedment technique for T-beams [58].

Even when using the NSM method, premature debonding can still occur, resulting in incomplete use of the tensile capacity of the common FRP repair material. This is more likely to happen in beams with T-shaped or I-shaped cross-sections. Additionally, studies have indicated that in the NSM method, detachment of the cover concrete in which the NSM reinforcement is used might occur which prevents the repair approach to work in full capacity. Therefore, it might not be possible to fully utilize the tensile strength of the repair material using the NSM or EB methods, unless proper anchorage is provided. This motivated another method which is embedding reinforcing materials well inside the girder, i.e., into the concrete core beyond the cover or surface grooves, to increase the bonding. This is because the concrete core handles the stress transfer to the strengthening material, and compared to the concrete cover, it can provide better confinement, and thus improved bond behavior. Additionally, in this deep embedment technique, protection against fire and vandalism is even more effective than the NSM method [30,57]. Figure 3 shows an example application of the deep embedded reinforcement method for RC T-beams.

### 2.4. Surface Preparation

Surface preparation, i.e., cleaning and roughening the surfaces of composites is a critical step in the repair process which can improve bond strength. An improperly prepared surface can result in debonding or delamination. Sandblasting, water jetting, grinding, brushing, air pressure, rounding of corners, pressure washing the concrete surface, surface patching, and nylon peel-ply techniques are commonly used for this purpose. Failure in proper surface preparation can result in damage to the repair material due the delamination of the concrete substrate [19,26,34,59,60,61]. The required steps for surface preparation are as follows:

#### 2.4.1. Removal of All Unsound Concrete

It is recommended to remove slightly more concrete rather than too little unless it affects the bond of prestressed strands. If patching is going to be done after unsound concrete removal, the chipped area should at least be one inch deep and should have edges as straight as possible, at right angles to the surface. Air-driven chipping guns or a portable power saw can be used for cutting the concrete. However, care should be taken not to damage the strands or the reinforcement [46].

#### 2.4.2. Select a Patching Method (If Needed)

**Figure 4:**
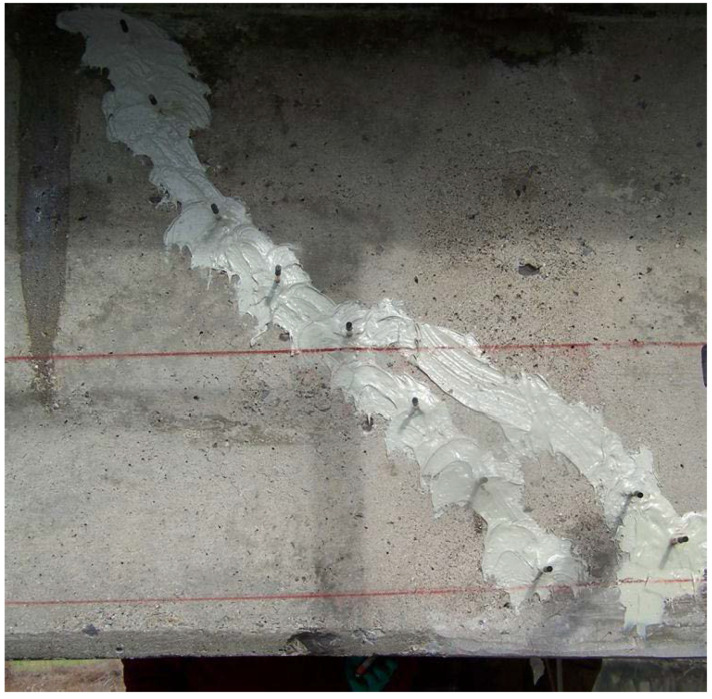
Example of RC girder crack filled with low-viscosity epoxy [63].

In case there are cracks on the girder, they should be filled with proper materials, i.e., patching. There are several patching methods and the most common five ones are discussed. (1) The dry pack method: which is suitable for holes having a depth nearly equal to the smallest dimension of the section, such as the core or bolt holes. The method should not be used on shallow surfaces or for filling a hole that extends entirely through the section or member. (2) The mortar patch method: which is appropriate for concrete members with shallow defects that require a thin layer of patching material such as in honeycombs, surface voids, or areas where concrete has been pulled away with the formwork. (3) The concrete replacement method: where the defective concrete is replaced with machine-mixed concrete that will become integral with the base concrete. This is preferred when there is a void extending entirely through the section, or if the defect goes beyond the reinforcement layer, or in general if the volume is large. (4) Synthetic patching: this method is beneficial where Portland cement patches are difficult or impractical to apply. Examples are patching at freezing temperatures or patching very shallow surface defects. In these situations, epoxy- and latex-based products can be used. Epoxies can be used for a variety of purposes such as bonding agent, binder for patching mortar, adhesive for replacing large broken pieces, or as a crack repair material. Small deep holes can be patched with low-viscosity epoxy and sand, whereas shallower patches require higher-viscosity epoxy and are more expensive. Although epoxies offer excellent bond and rapid strength development, they are hard to finish and usually result in a color difference between the patch and the base concrete. Therefore, it is suggested that epoxy mortars be used only in situations where exceptional durability and strength are required. Latex materials are used in mortar to increase its tensile strength, decrease its shrinkage, and improve its bond to the base concrete, thus helping to avoid patch failure due to differential shrinkage of the patch. Latex is especially useful in situations where feathered edges cannot be avoided. (5) Epoxy injection: this method is used to repair cracks or fill honeycombed areas of moderate size and depth. It becomes an important part of the repair process, specifically for corrosion-damaged girders in which cracking and spalling of the concrete is commonplace. Epoxy injection should be done only by appropriately trained personnel [46,62]. Figure 4 shows an example of a concrete girder surface after epoxy injection.

#### 2.4.3. Surface Polishing (Roughening)

As part of the surface preparation, the surface of the concrete is usually polished until fine aggregates are exposed [64]. This improves the bond between the main strengthening material and the concrete surface. Abrasive blasting or sand blasting is one way of surface roughening [65]. Diamond grinding is another technique utilized for this purpose [66]. It can also be done using high-pressure water jetting [67] or using a grinder where the roughening can be implemented to the aggregate level [17].

#### 2.4.4. Cleaning

The concrete surface should be cleaned before the application of the repair material. This can be done using a variety of methods including pressurized air and acetone or water jetting and pressure washing [64]. It is usually done using compressed air or water [51]. It can also be done using a wire brush. It is also important to make sure that the surface is dry and free from any oil, or greasy substances [68]. Sandblasting can also be used to clean the repair area [65]. Compressed air is also widely used for cleaning the concrete surface from dust and debris [60].

#### 2.4.5. Priming

In order to increase the performance of the repair that will be applied on the concrete substrate, a primer might be applied to the concrete surface. One example procedure is presented in [64], where a two-part primer is applied to the prepared concrete surface and left to be dried, then a two-part epoxy resin is applied to the primed concrete surface prior to the application of the FRP material.

### 2.5. Repair Design

Design of the repair is an important step to make sure that the chosen repair material is applied in a configuration that can provide sufficient strength and durability. Different design approaches might be required depending on the type of the damage, extent of the damage, the expected durability of the repair, availability of the resources for the application of the repair, etc. Additionally, design optimization techniques might be used to achieve the most efficient outcome [69]. In the case of using FRP-based materials for repair, the following codes can be consulted or adopted to provide recommendations on the repair design [34]:

- AASHTO Guide Specifications for Design of Bonded FRP Systems for Repair and Strengthening of Concrete Bridge Elements (latest version is 2013).
- AC125 Acceptance Criteria for Concrete and Reinforced and Unreinforced Masonry Strengthening Using Externally Bonded Fiber-Reinforced Polymer (FRP) Composite Systems (latest version is 2012). AC125 is issued by ICC Evaluation Service to establish minimum requirements for the issuance of evaluation reports on FRP composite systems under the 2012, 2009, and 2006 International Building Code (IBC) and the 1997 Uniform Building Code (UBC).
- ACI 440.3R Guide Test Methods for Fiber-Reinforced Polymers (FRPs) for Reinforcing or Strengthening Concrete Structures (latest version is 2004).
- ACI 440R Report on Fiber-Reinforced Polymer (FRP) Reinforcement for Concrete Structures (latest version is 2007).
- ACI SP-215 Field Applications of FRP Reinforcement: Case Studies (latest version is 2003).
- ISIS Design Manual No. 4, FRP Rehabilitation of Reinforced Concrete Structures, issued by the Canadian Network of Centers of Excellence on Intelligent Sensing for Innovative Structures (latest version is 2008).

### 2.6. Application of the Repair Material

The next step after surface preparation is the application of the repair material. Depending on the repair approach being used, i.e., EB technique, NSM method, or embedded reinforcement, the repair material should be applied in different ways and configurations. The process for the application of the repair material for each method is briefly described in this section, while the repair configuration, which mostly depends on the type of girder deficiency, is described in Section 3.2, Section 4.2 and Section 5.2, for shear, flexural, and fire damage deficiencies, respectively.

#### 2.6.1. EB Technique

**Figure 5:**
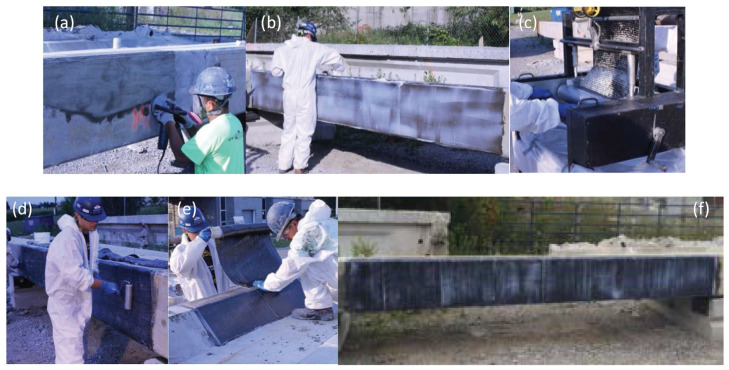
Illustration of wet layup for applying carbon fiber-reinforced polymer (CFRP) sheets for flexural strengthening: (**a**) surface preparation (**b**) surface primer application (**c**) FRP sheet saturation (**d**) longitudinal FRP layer (**e**) transverse FRP layer (**f**) final strengthening configuration (adopted from [70]).

The EB repair techniques using FRP materials are usually implemented in three ways: (1) wet layup, (2) pre-preg, or (3) pre-cured. In the wet layup approach (see Figure 5), the resin serves to both saturate the fibers and bind the sheet to the concrete surface. Dry fiber sheets are impregnated with a saturating resin on-site and bonded to the concrete substrate using the same resin to be cured. Usually the saturating and curing processes are done on-site, but they also might be implemented at the manufacturer’s facility off-site. This method has the advantage of the flexibility of the FRP sheets. Thus, it is appropriate for application on surfaces that are relatively smooth, but have an abrupt or curved geometry. The relatively smooth surface is a requirement here to make sure that a proper bond is achieved between the concrete and the strengthening material. Wet layup applications are suitable for column wrapping and U-wrap applications, but not recommended in general for flexural repair for prestressed concrete girders [1,19,28,46]. In the pre-impregnated or commonly referred to as pre-preg approach, the fiber sheets are saturated off-site and partially cured. On the site, they are bonded to the concrete surface using resin and they often require additional heating to complete the curing [19]. In the pre-cured approach, the resin is only used for gluing the procured (fiber and matrix already combined) laminates, strips, or sheets to the concrete surface. The fibers are saturated and cured off-site like precast concrete members. Pre-cured strips are available from a variety of manufacturers in discrete sizes and a number of grades. As for CFRP strips, high-strength (HS), high-modulus (HM), and ultra-high-modulus (UHM) grades are commercially available. In this method, the repair material is rigid and cannot be bent if a more flexible application is needed. Therefore, the application is limited to straight or slightly curved surfaces. This method is used when the surface of the structure is smooth and flat or when using the wet layup method is not practical [1,19,28,46,61].

#### 2.6.2. NSM Technique

**Figure 6:**
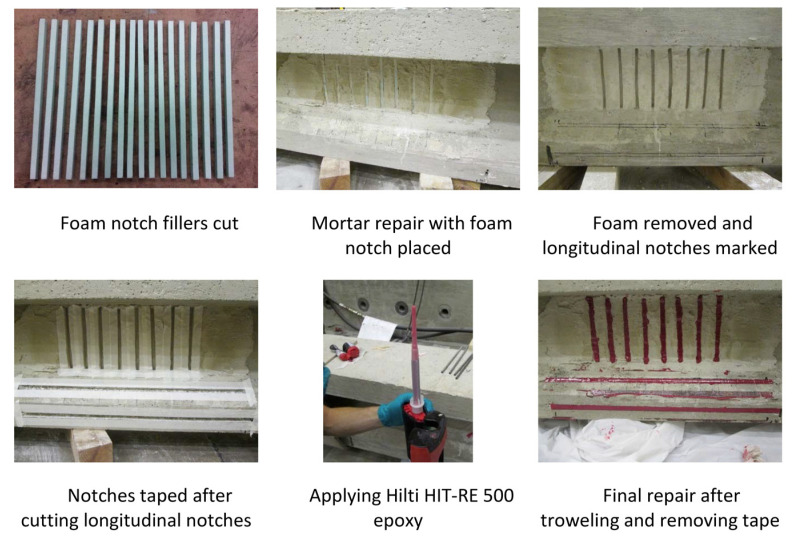
Example of the FRP NSM repair process [28].

First, grooves are made into the concrete surface, and the concrete in between the cuts is chiseled away. Then, the groove is cleaned, and dust is removed using compressed air. In order to have a clean final appearance, tape can be applied to the sides of the grooves. The strengthening material (bar, thin strip, etc.) is fastened into the groove using a filler material (epoxy resin, cement grout, etc.). Finally, the adhesive surface is leveled using a trowel and the tape is removed prior to the curing of the adhesive [28]. The procedure for an example application of NSM repair is shown in Figure 6.

### 2.7. Prestressing of the Repair Material (Optional)

**Figure 7:**
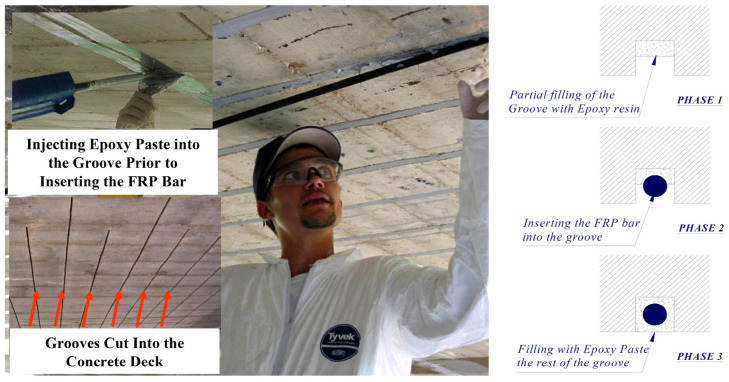
Implementation procedure for the prestressed NSM technique [72].

To increase the efficiency of the repair, the material for both EB and NSM methods can be prestressed. Prestressing was first utilized for strengthening bridges in the 1950s [55]. It enables the member to sustain higher loads and cover a longer span length due to the negative moment that is generated in the element. It is relatively fast and it can be done without impacting traffic [32]. It also helps to upgrade the performance of the member in terms of both load-carrying capacity and serviceability, e.g., controlled deflections and crack initiation, that could not be achieved otherwise [71]. Some of the advantages of prestressing the repair material are: fully utilizing the high strength of the material, improving the serviceability of RC beams, limiting the propagation of old cracks, delaying the formation of new cracks, enhancing the stiffness of the beams, better utilization of the strengthening material, smaller and better distributed cracks in the concrete, unloading (stress relief) of the steel reinforcement resulting in higher steel-yielding loads, and potential for the restoration of service level displacements or performance of the structure. Prestressing repair materials provides also a confining effect on the concrete and, significantly, any patch material because it places the concrete into compression, and in turn, causes a delay in the onset of cracking and a reduction in crack widths [31,56]. However, it should be noted that, generally, different levels of prestressed forces will result in different failure modes. Further, despite all the advantages of prestressing the repair material, the design of the end anchorage system requires accurate and expensive analysis due to the presence of large shear forces, large concentrated compressive forces, and induced moments due to the eccentric post-tensioning forces. If needed, the anchorage system should also be post-tensioned itself [19]. Figure 7 shows the prestressing setup and procedure for the implementation of the NSM technique for an RC girder.

### 2.8. Anchorage System

**Figure 8:**
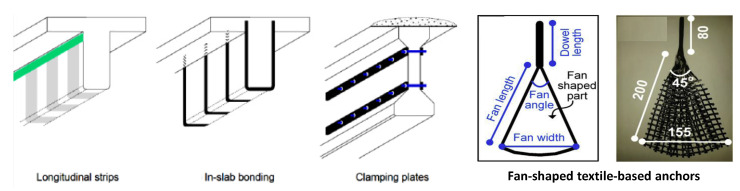
Schematic of the common anchorage methods (adopted from [7,34]).

**Figure 9:**
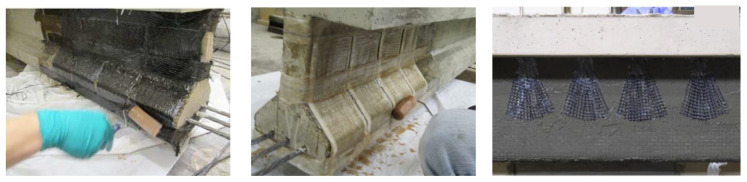
End anchorage system using CFRP or glass FRP (GFRP) strips [28], and plugs of fan-shaped CFRP anchors inserted into holes inside the concrete surface [7].

For cases of high peeling or shear stress, an anchorage system might be used in order to delay the debonding of the strengthening system such as FRP materials. A proper anchorage system might allow the use of a strengthening plan that otherwise would not meet the design code provisions, allowing the repair material to continue carrying load even after debonding occurs and thereby increasing its contribution. It can enable greater strengthening or the use of a wider range of possible configurations and material properties. Different anchorage systems have been introduced so far depending on the strengthening approach that they are used with. Some examples include: additional horizontal strips of the repair material, embedment of the repair material into the beam flange through precut grooves with adhesive bonding, various mechanical anchorage systems involving bolts and plates, and fan-shaped textile-based anchors [28,34,71,73]. Figure 8 shows a schematic of these systems. Moreover, Figure 9 shows real-life applications of the horizontal strips, which is the most common approach [28], as well as the application of fan-shaped textile-based anchors to the web–bottom flange interface of an RC girder [7].

The fiber-based anchors have the advantage of being light-weight and non-corrosive. Additionally, since the use of FRP-based or textile-based materials is commonplace for girder repair, using a compatible anchor material is also advantageous [7]. A drawback of the use of many anchorage systems is the added cost and complexity of installation [34].

### 2.9. Strand Splicing (If Needed)

**Figure 10:**
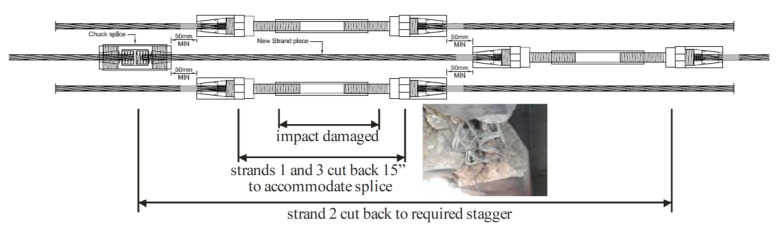
Staggering of the strand splices [15].

**Figure 11:**
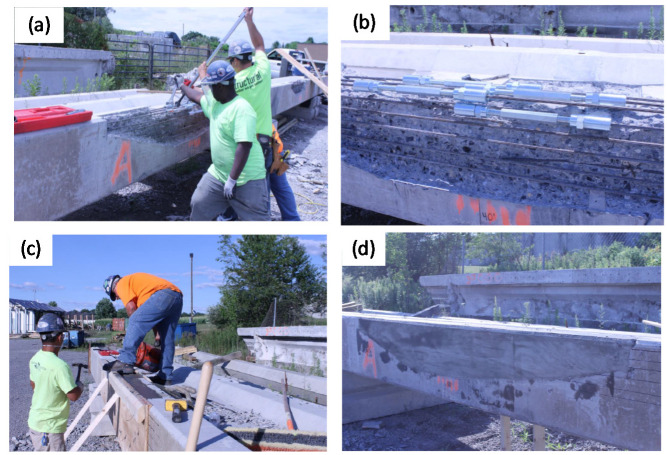
Repair procedure using strand splices: (**a**) installation of the strand splices, (**b**) completed installation of splices, (**c**) placing the repair concrete, and (**d**) completed repair after form removal (adopted from [19]).

When one or more prestressing strands in a prestressed girder are damaged, strand splicing can be used to do the repair. It is a fast, efficient, and cheap repair method for reconnecting damage or broken prestressing strands in order to restore the prestressing force. Strand splices alone cannot be relied on for fully restoring the ultimate strength of the strands or the element that is being repaired. That is because they are limited to developing 85% of the nominal strength of the strands they are joining (0.85 *fpu*). In order to increase their efficiency, the splices should be staggered as illustrated in Figure 10 and limited to splicing 15% of strands in a girder, regardless of staggering [74,75]. It should be noted that commercially available splices are available for strand diameters only up to 0.5 in [19,46,74]. Additionally, strand splices are internal applications and therefore may be used with almost any external application. The NSM method might be an exception since interference between the strand chucks and NSM slots might happen [46]. However, they can be combined with an externally bonded repair method using a repair material such as FRP or FRCM [38]. Figure 11 illustrates the procedure for the strand splice repair of an RC girder.

## 3. Repair for Shear

One of the requirements of the current codes in the assessment of old RC bridges is evaluating their shear capacity. In the following sections, the main reasons for the shear deficiency of RC bridge girders and common solutions are summarized and followed by recommendations for the repair procedure.

### 3.1. Main Causes of Damage

**Figure 12:**
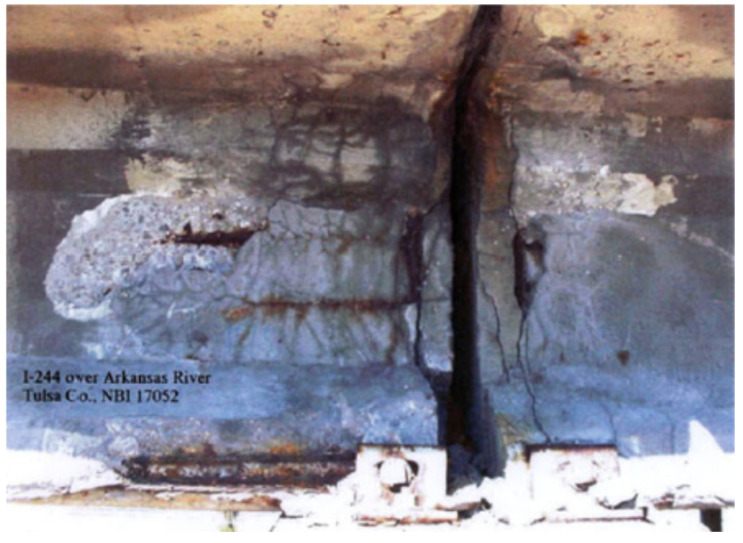
Damage in the end region of an AASHTO bridge girder [21].

Shear deficiency of RC girders can be caused by an insufficient amount of shear reinforcement, low concrete strength and/or increased design load, and corrosion of existing shear reinforcement [7,8]. Corrosion of the shear reinforcement is the main reason for shear deficiency and is mainly caused by corrosion at girder ends which can expose the shear reinforcement. The end regions of the girders are more susceptible to corrosion due to the proximity to the deck joints which exposes them to the seepage and chlorides from deicing salts in cold climates. Since the 1960s, when officials started applying deicing salts in the winters to bridge structures, the deterioration rate of concrete girders has increased significantly. This has caused both economic and technical issues for bridge structures. On the other hand, the shear demand of the girder in the end regions is the highest, making the situation critical. This becomes even more critical for prestressed girders since the load is transferred to the beam through a bond between the prestressing strands and the concrete in the end zones of such girders, causing even more shear demands. High potential for corrosion together with high shear demands makes the girder end regions in need of special care in terms of shear capacity requirements. Other situations that can make the shear deficiency of the girder ends more crucial include: (1) failure of the expansion joint that can cause all of the deicing salts to drain over the girder end, (2) a partially fixed girder end, such as one created by a frozen bearing, may impose additional stress at the girder end and when the build-up stress is relieved, the girder might crack in tension or in shear, and (3) when the beams are transversely post-tensioned in the horizontal plane and made contiguous within a deck. This is a common typology of bridge girders used for railways under bridges, with simply supported spans ranging from 6 to 20 m [2,21,28,57]. Figure 12 shows an example of a typical damage at the end of a standard AASHTO girder.

### 3.2. Common Solutions

The chosen repair material for shear repair can be applied to the damaged girder in any of the three methods described in Section 2.5, i.e., EB or NSM or the deep embedment method. The possible configurations for each of these three methods are explained next.

#### 3.2.1. EB Technique

**Figure 13:**
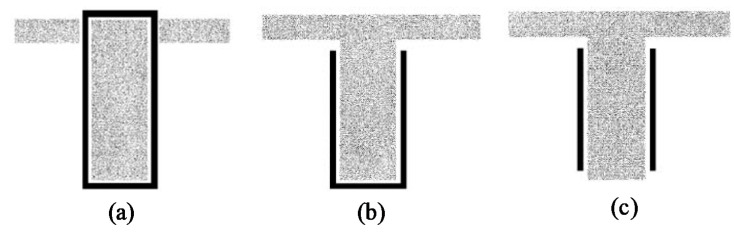
Wrapping schemes for EB FRP shear laminates: (**a**) complete closed wrapping, (**b**) 3-sided U-wraps, and (**c**) 2-sided face plies [28].

If this method is used for shear repair, it might be used in the three following common forms: complete wrapping, three-sided U-wraps, and two-sided face plies as illustrated in Figure 13. The full wrapping approach is the most efficient shear strengthening scheme. This is because it is capable of achieving the failure mode of the repair material and, in turn, utilizing the material’s full strength. However, debonding most likely occurs first. Standards and design guidelines from ACI and AASHTO, for instance, recommend the use of closed wrapping in beams whenever possible. However, most RC beams are cast monolithically with slabs, and therefore the technique is rarely adopted in the field. U-wrap is popular in practice because of its wide applicability and ease of installation. Nevertheless, most U-wraps and almost all the two-sided retrofits result in a debonding mode of failure with very little ductility. In these cases, anchoring the fibers, preferably in the compression zone, can be used to increase the effectiveness of the system. Properly designed anchors can result in the repair material reaching its tensile capacity prior to debonding like in a full-wrap system. Additionally, it should be noted that in most T-beams, the neutral axis occurs within the depth of the flange. While the U-wrap terminates below the flange, the anchorage region of the U-wrap is thus located below the neutral axis, i.e., in the tension region of the beam. This also means that the tension and compression regions of the beam will not be connected by the wrap [8,28,34,51,57,73].

**Figure 14:**
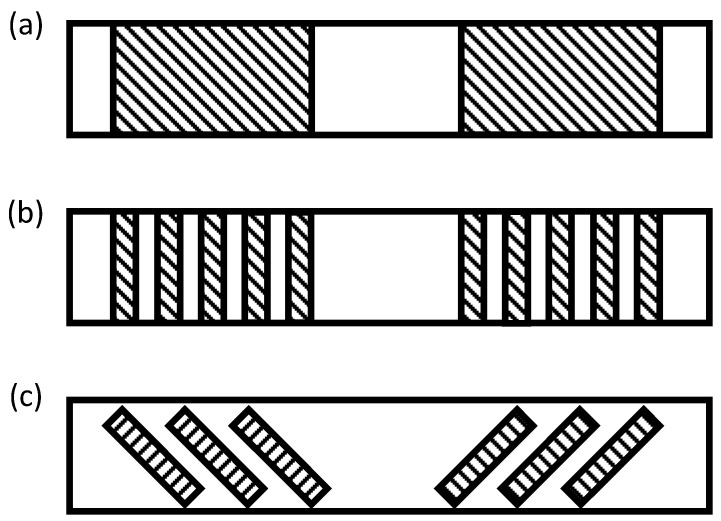
Different strengthening schemes: (**a**) Continuous wraps (**b**) discontinuous vertical strips (**c**) discontinuous inclined strips.

Any of the three EB configurations shown in Figure 13 and described above can be implemented with different details. They can be applied continuously or discontinuously along the length of the girder (see Figure 14). In the case of the discontinuous application, the spacing between the strips becomes important. The strengthening strips can also be applied vertically or inclined to the surface of the damaged girder as illustrated in Figure 14. Continuous or discontinuous application of the repair material, spacing of the repair strips in the case of discontinuous application, and the inclination of the repair strips can significantly affect the effectiveness of the repair and the mode of failure of the repaired girder, and are thus considered important design parameters.

#### 3.2.2. NSM Technique

The NSM technique is used for the shear repair of RC bridge girders by making vertical or inclined grooves filled with a proper repair material on the web of the damaged girder, as illustrated in Figure 6 shown above. Similar to the EB method, the inclination of the grooves as well as the spacing between them are important design considerations.

#### 3.2.3. Deep Embedment Method

**Figure 15:**
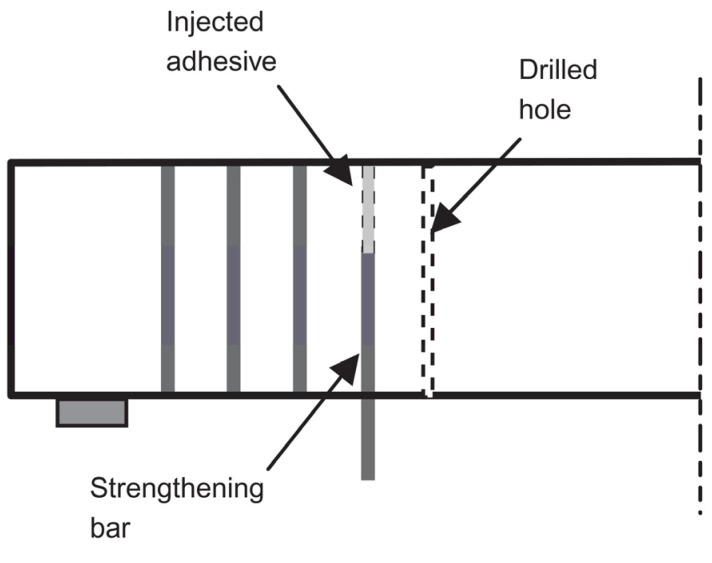
Shear strengthening using deep embedment techniques [57].

This method is mainly used for the shear strengthening of girders, especially where access to the girder web is not possible [57]. In this method, as explained in Section 2.3, vertical or inclined holes are drilled into the concrete section, in the shear zone, upwards from the soffit as shown in Figure 15. The bond between the strengthening bars and concrete is achieved using epoxy resin.

### 3.3. Recommendations

This section provides recommendations for the repair of girders with shear deficiency, based on the repair case studies found in 62 different studies, including both research and real-life applications of the repairs which are both critical for achieving a sufficient understanding of a repair technique. At less intense levels, cracking can affect the serviceability and durability of the girders which might be treated using an appropriate method such as coatings, sealers, overlays, electrochemical methods, corrosion inhibitors, admixtures, patching, reinforcing steel protection, and membranes. Protective coatings, most of which contain an epoxy resin system, as well as penetrating or surface sealers are the most popular repair approaches for such low-intensity damage levels. Higher levels of damage, i.e., structural deficiencies, require implementation of an appropriate repair approach. Shear repair of structurally deficient girders usually involves proper treatment of the steel reinforcement, restoring the shape of the section using mortar or concrete which can include corrosion inhibitors, injection of the cracks with a proper material such as epoxy, and finally, surface preparation and the application of the main repair material.

**Figure 16:**
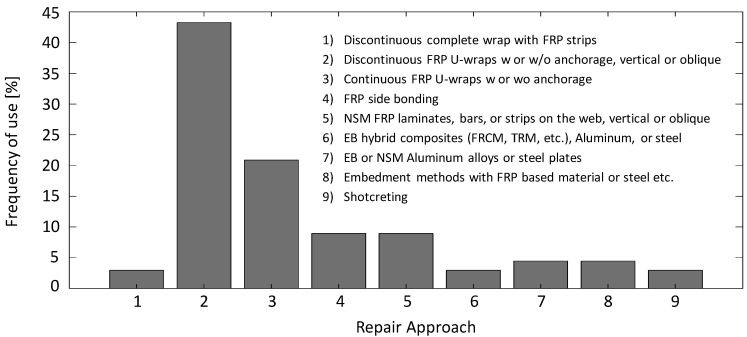
Summary of shear repair techniques of cracked RC girders and frequency of use in the literature.

For a complete discussion of implementation techniques of the main repair material, all the different repair techniques found in the literature are summarized and grouped in Figure 16 to indirectly rank which methods are more popular or common. The methods summarized in the figure along with the respective studies or references are: (1) discontinuous complete wrap with FRP strips [67,76]; (2) discontinuous FRP U-wraps with or without anchorage, vertical or oblique [3,10,12,14,28,47,51,62,64,66,73,77,78,79,80,81,82,83,84,85,86,87,88,89,90,91,92,93,94]; (3) continuous FRP U-wraps with or without anchorage [8,21,62,68,78,95,96,97,98,99,100,101,102,103]; (4) FRP side bonding [12,102,104,105,106,107]; (5) NSM FRP laminates, bars, or strips on the web, vertical or oblique [28,85,108,109,110,111]; (6) EB hybrid composites (FRCM, TRM, etc.), aluminum, or steel [3,7]; (7) EB or NSM aluminum alloys or steel plates [9,21,112]; (8) embedment methods with FRP-based materials, steel, etc. [30,58,68]; and (9) shotcreting [113,114].

As seen from Figure 16, the most utilized shear repair method is FRP U-wraps. While discontinuous U-wraps (installed vertically or obliquely) are the most common approach, continuous U-wraps have also been used quite extensively. However, Mofidi and Chaallal [78] indicated that there is no need for using additional material for continuous U-wraps or side-bonded sheets since the discontinuous wraps were shown to be more effective in increasing the shear capacity, but they come at a price of relatively increased deflections. The use of discontinuous wraps also provides a better condition for future visual inspection of the repair performance. Moreover, the width, thickness, spacing, and inclination of the FRP strips are other design parameters that affect the performance of the repair. Mofidi and Chaallal [78] and Qapo, Dirar [10] indicated that wider strips or higher width-to-spacing ratios contribute more to the shear capacity. Increasing the thickness was also shown to enhance the shear capacity [10]. Kang and Ary [79] reported an increase in strength and ductility when the spacing of the FRP strips was less than half the effective depth of the beams, while larger spacings hardly improved the behavior. As for the inclination of the strips, while the inclined repair schemes are expected to be more effective, the labor for their installation is also expected to be more. Thus, the repair material orientation should be specified based on the specific project requirements and the tradeoff between the labor and the efficiency of the repair.

**Figure 17:**
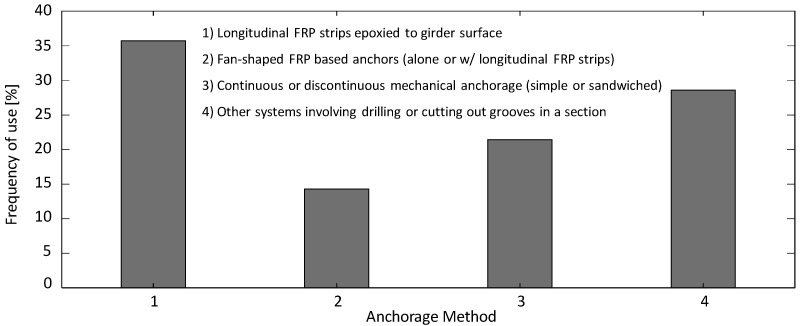
Frequency of use of different anchorage systems found in the literature.

As for the anchorage system that might be used in conjunction with the main shear repair method, the different methods and their frequency of use as found in the literature are summarized in Figure 17. Four methods are found: (1) longitudinal FRP strips epoxied to the girder surface [28,47,73,77,83,84]; (2) fan-shaped FRP-based anchors (alone or with longitudinal FRP strips) [3,115]; (3) continuous or discontinuous mechanical anchorage (simple or sandwiched) [47,73,77]; and (4) other anchorage systems involving drilling or cutting out grooves in the section [8,51,84,94]. Figure 17 shows that the longitudinal FRP strips are the most common utilized approach, but in general, there is not a significant difference between the frequency of use of the methods. Accordingly, it can be inferred that there is no single dominant anchorage system that can be applied to the majority of shear repair projects. Additional horizontal FRP strips are very easy to install and require the least amount of labor among all anchorage systems. Yet, different levels of effectiveness have been observed in various studies [47,73]. The mechanical anchorage systems have shown good performance in some cases but can cause damage to the FRP material [115]. This is where the fan-shaped FRP-based anchors can be useful. Other anchorage systems involving drilling or cutting out grooves in the section such as in-slab bonding have also been proposed in the literature. However, the authors believe that, in the case of spending money and labor work in the complex installation on-site such as cutting grooves, the NSM techniques can provide a more efficient way of repair compared to an EB method with a complex anchorage system.

Although the NSM methods require more labor for their implementation, they usually result in less material use. They also have better bond behavior in general, which usually leads to a higher capacity increase as a result of the full utilization of the FRP material. The quality of the concrete inside the groove is typically superior to the surface concrete, which adds to the efficiency of the repair. Further, surface preparation is minimized in NSM and such methods exhibit better resistance to corrosion and improve serviceability. Such reasons might justify and offset the NSM methods’ additional initial costs as compared to EB methods. It is also noted that in shear repair applications, the repair procedure is usually implemented on the web of the girder. Therefore, most likely, there will be no need for above-head groove cutting or other highly inconvenient practices. Moreover, the grooving is obtained with a single saw cut without any concrete chipping. Thus, instead of using complex, labor-intensive, and expensive anchorage systems in conjunction with the EB U-wraps that seem to be the common shear-strengthening approach at the time, NSM methods can be used for an improved structural performance and higher long-term economy.

**Figure 18:**
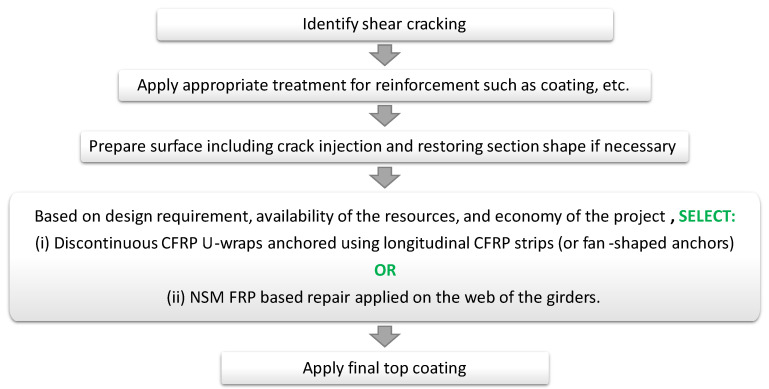
Recommended repair procedure for RC bridge girders with shear cracks.

To summarize, EB FRP U-wraps with longitudinal FRP strips used for anchorage are the most common and well-researched technique for the shear repair of RC bridge girders, which can usually increase the shear capacity of the girders at least by 25%. In the case that a higher increase in the shear capacity is needed, use of fan-shaped FR-based anchors in conjunction with longitudinal strips seems to be a promising approach that has gained popularity in recent years. However, if the given project conditions as well as human and monetary resources allow for the implementation of the NSM technique, then it is recommended to consider an NSM method. The long-term efficiency is proven to be superior in the case of NSM as compared to the EB method. The procedure for RC girders shear repair with the above recommended two methods is presented in a simple flow chart and given in Figure 18 for the convenience of readers and future use.

## 4. Repair for Flexure

In this section, the main causes of damage to the flexural capacity of RC girders are described, followed by a discussion of common solutions found in the literature and recommendations for the repair procedure based on surveying the frequency of use and authors’ suggestions.

### 4.1. Main Causes of Damage

Material ageing and deterioration, inadequate reinforcements, change in usage, and overloading of the structure are the main reasons for flexural deficiency of reinforced concrete structural elements [27]. Overheight vehicle impact, even though it does not generally cause immediate collapse of the bridge, can result in further or accelerated deterioration often resulting in significant prestressing steel corrosion which can lead to major flexural deficiency. Overheight vehicle impact happens when a vehicle’s, most commonly large trucks, height is greater than the vertical clearance between the roadway and overpass and the vehicle strikes the overpass [116]. Although an accurate record of the number of overheight vehicle impacts is not available in the literature, it is estimated that about 1100 of such collisions happen yearly in the US [19], which proves the importance of the topic. According to ElSafty, Graeff [117] and Gangi [74], vehicle collision happens in the US 25 to 35 times per year and per state. In 2008, it was reported that just in the state of New York, 32 bridges had been impacted a total of 595 times since the mid-1990s [24]. Fu, Burhouse [118] indicated that the frequency of overheight accidents reported in Maryland increased by 81% between 1995 and 2000. Further, an analysis of the statewide accident database showed that of the 1496 bridges susceptible to impact by overheight vehicles statewide, 309 (20%) had been struck, with 58 (4%) requiring repairs. As of 2011, Texas DOT has repaired more than 30 impact-damaged concrete bridges using CFRP materials [65]. Due to the high frequency and high intensity of flexural damage due to vehicle collision, this section will focus on the reported repair techniques that are mainly utilized for vehicle impact damage.

**Figure 19:**
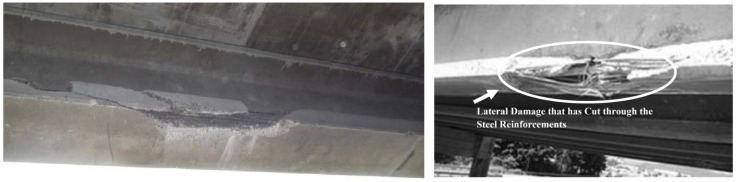
Severe concrete spalling due to overheight truck impact damage [6], and example of lateral impact damage on steel reinforcement [24].

Vehicle impact can cause damage to the girder concrete cover or cut through the steel reinforcement and/or prestressing cables [24]. Figure 19 shows an example of impact damage with two different severities: damage to the concrete cover, and damage to the steel reinforcement. In general, impact damage does not cause immediate collapse of the structure. However, when untreated, it can result in further or accelerated deterioration often resulting in significant prestressing steel corrosion [116]. It would be best to prevent any collision damages to occur, but it is important to have practical, quick, and cost-effective repair schemes in case overheight vehicle impacts occur [19]. The following section identifies common methods for the repair of bridge girders that have flexural deficiencies due to vehicle impact damage or other causes.

### 4.2. Common Solutions

Bridge girders experiencing impact damage are usually subjected to: (1) concrete crushing, (2) prestressed strand and/or steel reinforcement being exposed which makes them more susceptible to further damage and corrosion, and (3) prestressed strand loss. Harries, Kasan [119] indicated that when 25% of the strands in a girder no longer contribute to its capacity, girder replacement is a more appropriate solution. Otherwise, for the purpose of the repair of the section and restoring its capacity, the severed prestressing strands are usually spliced together, and the shape of the section is restored by concrete or mortar. Cracks, if any, are filled with a proper available material such as epoxy. Surface preparation is implemented, ready for the main repair material to be applied. Common methods of the application of the repair material to the girders for flexural repair are mostly through EB and NSM techniques. Details of the commonly used configurations for each of these techniques are explained next.

#### 4.2.1. EB Technique

**Figure 20:**
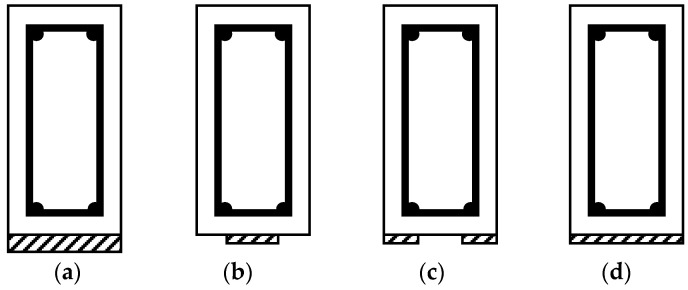
CFRP laminates for the flexural strengthening of girders: (**a**) two-layer sheet (**b**) one-layer strip (**c**) one-layer strips (**d**) one-layer sheet.

For flexural repair using the EB technique, it is often recommended that the repair material is applied to the soffit of the girder since the material near the neutral axis is less efficient in strengthening the element [75]. This is considered as the earliest and most basic method for upgrading and retrofitting the beams in the flexure [25]. Extending the repair material vertically up the web will reduce the efficiency and does not affect the ultimate debonding limit state [56]. Figure 20 shows three different EB configurations of FRP laminates with different widths attached to the bottom of the girders through epoxy adhesives for repair or strengthening purposes. The EB configurations shown in Figure 20 are usually used in conjunction with transverse wraps for enhancing the bond behavior between the repair material and the girder.

#### 4.2.2. NSM Technique

**Figure 21:**
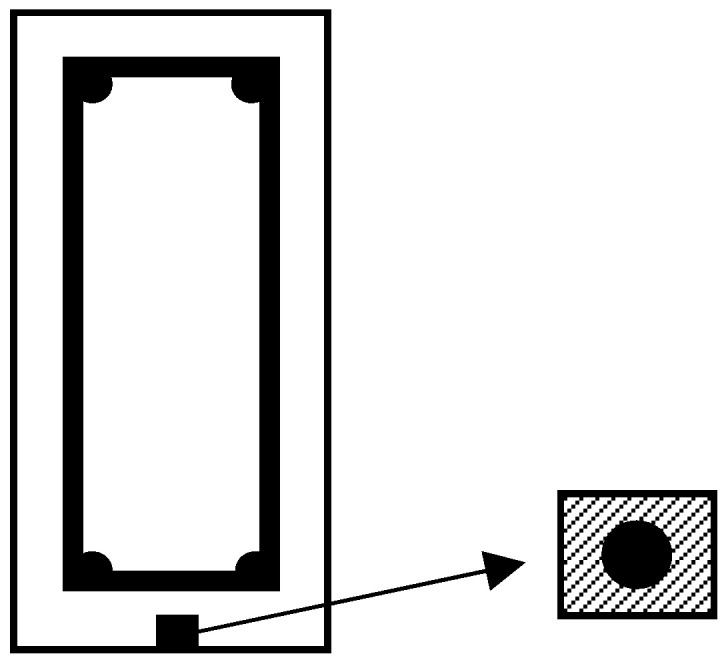
Schematic of application of prestressed NSM rods in cross-section (**left**) and inside the groove (**right**) for the flexural strengthening of rectangular beams.

In this method, similar to the flexural repair using the EB technique, the repair material should be applied to the soffit of the girder through grooves to have the best efficiency. Figure 21 shows an example for a repair configuration using NSM techniques.

### 4.3. Recommendations

**Figure 22:**
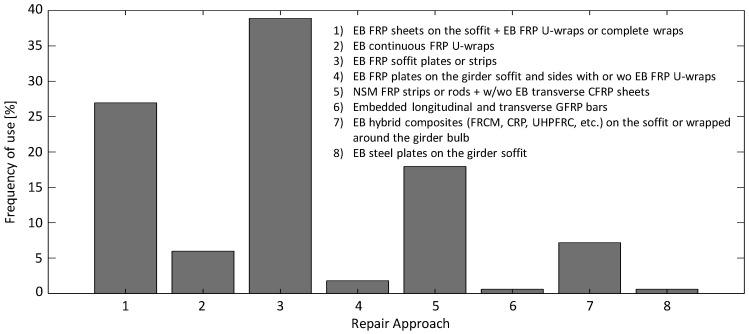
Frequency of use of flexural repair techniques of damaged RC girders in the literature.

This section provides recommendations for the repair of girders with flexural deficiency, based on the repair case studies found in 151 studies, including both research and real-life applications of the repairs which are both critical for achieving a sufficient understanding of a repair technique. Eight different methods were found in the literature: (1) EB FRP sheets on the soffit + EB FRP U-wraps or complete wraps [24,46,65,67,75,76,89,101,102,103,111,117,120,121,122,123,124,125,126,127,128,129,130,131,132,133,134,135,136,137,138,139,140,141,142,143,144,145,146,147,148,149,150,151,152]; (2) EB continuous FRP U-wraps [17,38,60,65,70,103,153,154,155,156]; (3) EB FRP soffit plates or strips [27,31,39,67,101,111,121,122,133,144,149,157,158,159,160,161,162,163,164,165,166,167,168,169,170,171,172,173,174,175,176,177,178,179,180,181,182,183,184,185,186,187,188,189,190,191,192,193,194,195,196,197,198,199,200,201,202,203,204,205,206,207,208,209,210]; (4) EB FRP plates on the girder soffit and sides with or without EB FRP U-wraps [63,126,211]; (5) NSM FRP strips or rods with or without EB transverse CFRP sheets [16,35,36,50,53,54,59,71,125,135,139,140,145,157,176,181,182,189,195,212,213,214,215,216,217,218,219,220,221,222]; (6) embedded longitudinal and transverse GFRP bars [6]; (7) EB hybrid composites (FRCM, CRP, UHPFRC, etc.) on the soffit or wrapped around the girder bulb [17,19,29,33,38,40,41,70,176,223,224,225]; and (8) EB steel plates on the girder soffit [204]. Figure 22 shows the frequency of use of those different repair approaches in the literature. The most utilized repair method for a flexural deficiency in girders is FRP plates or strips externally bonded to the girder soffit. It can also be seen that EB techniques, with or without transverse wraps, have been used more frequently compared to the NSM techniques. The reasons for this are the relatively easier implementation of EB methods, lower costs, and their ability to act as additional sacrificial reinforcement to prevent damage due to the future potential impacts. However, upon the availability of the resources (e.g., equipment) and expertise, the implementation of an NSM technique could be more suitable than an EB method. This is because the NSM technique, in general, can lead to a higher increase in the girder capacity due to the enhancement in the bond behavior which enables the girder to take advantage of the full capacity of the repair material. Further, the corrosion resistance of the repair material is better compared to EB techniques due to the placement of the repair material inside grooves in the cover concrete. Additionally, the NSM technique usually uses less repair material, which might provide some economic benefits for initial costs and long-term costs.

The most common anchorage system found in the literature is transverse U-wraps evenly spaced along the entire length of the girder or at parts where it is necessary. The U-wraps enhance the bond behavior of the FRP sheet attached to the tension side of the girder as a means of flexural strengthening. They also help in reducing crack propagation in the concrete section. While continuous CFRP U-wraps are also common for the repair of impact-damaged girders, Graeff [24] showed that the performance of the continuous U-wraps, in the absence of shear deficiencies, is not enhanced over evenly spaced discontinuous U-wraps. In the situations where shear strengthening of the section is also required, continuous FRP U-wraps or discontinuous wraps on required regions of the girders (such as shear spans) with appropriate spacing might be used.

**Figure 23:**
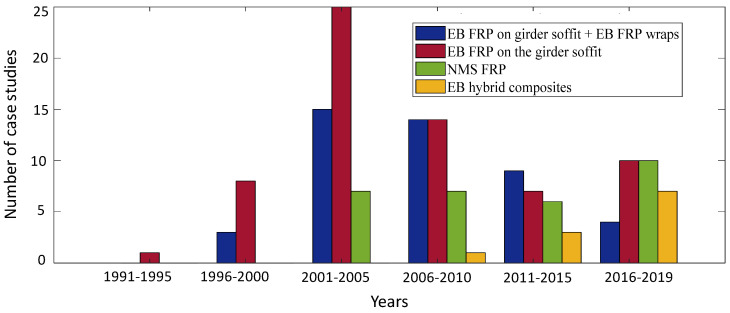
Evolution of case studies of the most common flexural bridge girder repair approaches over the past three decades.

The most commonly used material for the repair of impact-damaged girders seen in the literature is CFRP in the form of sheets and strips. However, as mentioned before, the choice of the repair approach, including the repair material and repair adhesive, highly depends on the specific project requirements and available resources. Figure 22 shows that the use of hybrid composites has also been quite frequent for the flexural repair of girders. This can be beneficial due to the enhanced properties of such materials compared to ordinary FRP, including improved ductility. The four most common repair approaches out of the eight methods shown in Figure 22 are also tracked over time to find their frequency of use over five-year increments starting in 1991 and up to 2019, as illustrated in Figure 23. The 2001–2005 range is shown to be the popular timeline for study and applying flexural repair methods for RC bridge girders. It can also be seen that FRP soffit bonding, with or without FRP wraps, is the most well-researched method of repair for flexures within that time range. It can be inferred from Figure 23 that the NSM FRP method and the EB hybrid composites are the two emerging methods since 2005. It is evident that in spite of the advantages that these two methods might have, their use will most likely involve more uncertainties compared to the well-studied FRP soffit bonding methods.

**Figure 24:**
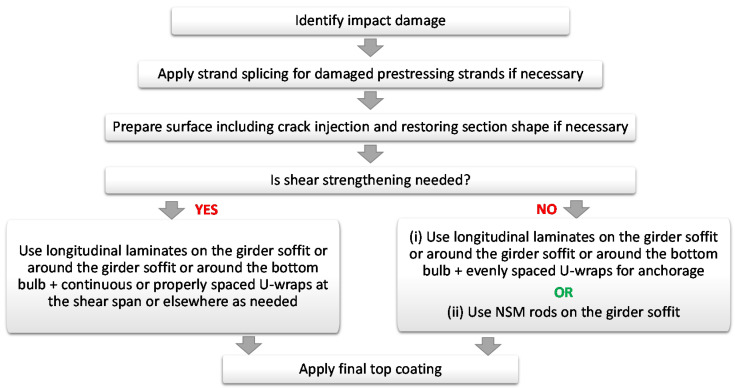
Recommended repair procedure for RC bridge girders with impact or flexure damage.

Based on the previous discussion, the recommended repair process for flexural or impact damage is presented in Figure 24, which considers whether shear strengthening is needed for completeness. The recommendation is to use longitudinal laminates EB to the girder soffit in conjunction with evenly spaced U-wraps as anchorage, where shear strengthening is not needed, or in conjunction with properly spaced U-wraps, where shear strengthening is required. If the project conditions allow and no shear strengthening is necessarily needed, NSM mounted rods on the girder soffit might be used. As for the repair material, the most popular repair material was found to be CFRP, but there is flexibility in the choice of the material depending on the specific project conditions.

## 5. Repair for Fire Damage

### 5.1. Main Causes of Damage

Fire hazards for bridges are caused by crashing vehicles, burning of fuels in the vicinity of the bridge, arson, and wildfire [48]. Fire damage is rare, but occurs occasionally when the resulting elevated temperature is high enough to damage the concrete cover. The heat dehydrates the concrete, evaporating its stored pore water, which weakens the cover concrete and reduces its compressive strength. This reduction can be up to 70% with a further increase in the temperature. The heat may also result in cracking, delamination, and spalling from the expansion of aggregates and steel reinforcement. Where external FRP wraps are used for the strengthening of bridge girders using epoxy resin as the adhesive, it is important to acknowledge that the mechanical properties of the epoxy resin are influenced by the temperature and that they significantly degrade at or above the glass transition temperature. At this temperature, the resin changes from a glassy state to a viscoelastic state. This is why ACI 440 recommends ignoring the capacity contribution of the FRP in such situations. Thus, bridge hydrocarbon fire hazards in FRP retrofit projects should be considered as an important factor in the repair design in case the bridge is identified as fire-critical. This is important due to the substantial increase in petrochemical transport along the nation’s vast highway network and the high number of bridge collapses caused by fire which cause extreme economic impact. The collapse of the two-span MacArthur Maze Bridge in Oakland, California, on April 29, 2007 due to a fire is an example, which caused an estimated USD 6 million/day total economic impact to the Bay Area [48,49,61].

### 5.2. Common Solutions

High temperatures resulting from a fire hazard can adversely affect the performance of epoxy resins. Therefore, in the case of considering FRP-based repair and epoxy resin materials for a bridge, ACI 440 recommends ignoring the capacity contribution of the FRP. This is a major issue in the utilization of the most common materials, i.e., FRP materials, in the repair of bridge structures. Two proposed solutions to this problem are: (1) the use of cement-based adhesives instead of epoxy, which has been exhibited in part of the shotcrete repair of a bridge in Texas after intensive fire damage [65]; or (2) application of cement-based fireproofing to the FRP layers as demonstrated in a research study by Beneberu and Yazdani [49].

### 5.3. Recommendations

Based on the brief discussion above, if an appropriate repair option is needed for a bridge prone to fire hazard or a fire-critical bridge, one of the following three recommended approaches can be considered: (1) use of FRP-based repair with a cement-based adhesive instead of epoxy; (2) use of FRP-based repair with a cement-based fireproof coating; or (3) use of cement-based repair such as shotcrete with or without complementary FRP repairs such as confining FRP U-wraps.

## 6. Concluding Statement

Bridge girder repair is an important topic due to the aging of bridge structures in the US and because full girder replacement is not favorable due to the associated higher costs and longer downtime causing traffic shutdowns and inconvenience. The goal of this study is to provide a comprehensive reference for researchers, engineers, and decision makers to compare different repair approaches for RC bridge girders, and find the best method that suites their specific repair problem to provide the highest economy, efficiency, and safety. Three main girder deficiencies in bridge structures are identified and discussed herein, which are shear deficiencies due to corrosion and exposure of the shear reinforcement, flexural deficiencies due to vehicle impact damage, and fire damage.

The choice of a repair approach is highly dependent on the specific conditions of a project (i.e., type of bridge, type and extent of the damage, available resources, etc.). However, the following methods were found to be the most common and efficient means of repair, and hence recommended in case a quick decision is needed. For shear repair, either EB CFRP U-wrap (continuous or discontinuous, vertical or oblique) or NSM CFRP laminates on the web with no need for anchorage or above-head installation can be used. For flexural repair, EB CFRP sheets on the soffit can be used in addition to EB discontinuous CFRP U-wraps. For fire repair, the use of cement-based adhesives instead of epoxy and/or fireproofing of FRP layers is recommended.

## Figures and Tables

**Table 1:** Damage classification of bridge components and structures.

Damage Classification [15]				
Minor	Moderate	Severe		
		Severe I	Sever II	Severe III
Damage does not affect member capacity	Damage does not affect member capacity	Requires structural repair	Requires structural repair	Damage is too expensive
Repairs are for preventative or aesthetic purposes	Repair is done to prevent further deterioration	Repair is done to restore ultimate limit state	Repair is done to restore both the ultimate limit state and the service limit state	The member must be replaced
